# Mineral-microbiota interactions in aquaculture: implications for fish gut health and nutrition

**DOI:** 10.1007/s11259-026-11207-2

**Published:** 2026-04-13

**Authors:** Fatmagün Aydin, Şehriban Çek

**Affiliations:** 1 Biotechnology Research and Application Center, Cukurova University, Adana, 01330 Türkiye; 2 Faculty of Marine Science and Technology, Iskenderun Technical University, İskenderun, Hatay 31200 Türkiye

**Keywords:** Sustainable aquaculture, Microbiome, Trace minerals, Mineral bioavailability

## Abstract

Dietary minerals and gut microbiota engage in a dynamic, bidirectional relationship that shapes the health, immune competence, and productive performance of farmed fish and shrimp. This review explores the bidirectional interactions between mineral supplementation and microbial communities within the gastrointestinal tract of farmed fish and examines the effects of individual and combined mineral supplementation including iron, zinc, magnesium, selenium, manganese, and copper in inorganic, organic, and nanoparticle forms on the intestinal microbiota and histomorphology of farmed aquatic species. Minerals serve essential physiological roles while simultaneously modulating microbial diversity, composition, and metabolic activity; conversely, the gut microbiota influences mineral bioavailability and absorption through enzymatic transformations and competitive uptake. Studies conducted on yellow catfish, largemouth bass, golden pompano, grouper, Nile tilapia, Chinese tongue sole, Pacific white shrimp, channel catfish, zebrafish, and Oriental river prawn were comprehensively examined. Findings indicate that organic and nanoparticle mineral forms generally exhibit higher bioavailability and more favorable effects on intestinal health compared to conventional inorganic sources, with partial substitution strategies (e.g., ~ 50% organic mineral replacement) yielding optimal outcomes in combined formulations. Optimized mineral supplementation was further associated with enrichment of beneficial microbiota, enhanced mucosal barrier integrity through goblet cell proliferation, and reinforcement of innate immune responses, collectively supporting nutrient assimilation, growth performance, and disease resistance. However, the reviewed studies share critical limitations: species diversity was narrow, experimental durations were short (8–80 days), no trial encompassed a full reproductive cycle, and the mechanisms underlying mineral–microbiota crosstalk remain incompletely understood. Synergistic or antagonistic interactions among Zn, Cu, Mn, Fe, and Se are inadequately characterized, and dose optimization specific to species, age, and physiological status has not been achieved. Future research should incorporate long-term and multigenerational designs, metagenomic and metabolomic analyses, comparative multi-mineral trials, and the integration of microbiome-based diagnostics to tailor mineral interventions, alongside validation under commercial aquaculture conditions and ecotoxicological assessment of nanoparticles in aquatic environments.

## Introduction

Aquaculture is expanding rapidly to meet rising global protein demand, yet the relationship between dietary minerals and gut microbiota remains a critically underexplored area with profound implications for fish health, nutrition, and the sustainability of global fish production. Understanding gut ecosystem interactions has become essential for optimizing production efficiency and animal welfare (Lall and Kaushik 2021; Kilercioglu et al. 2023; Luan et al. 2023; Kabir et al. 2025).

Minerals serve dual roles in aquatic organisms: they are essential nutrients for physiological processes such as enzyme activation, osmoregulation, skeletal development, and immune function, while also acting as modulators of gut microbial communities (Watanabe et al. 1997; Huang et al. 2025). The gastrointestinal tract hosts diverse microbial populations that contribute to digestion, synthesis of essential compounds, pathogen exclusion, and immune modulation. These microorganisms interact continuously with their chemical environment, including mineral composition.

Emerging evidence shows that dietary minerals influence microbiota composition, diversity, and metabolic activity, while the microbiota affects mineral bioavailability and absorption through enzymatic transformations such as phytase activity and microbial iron reduction and competitive uptake (Zafar and Khan 2020; Mmanda 2025). Certain minerals selectively promote beneficial bacteria while inhibiting pathogens, directly impacting disease resistance and growth. Microbial metabolism can also transform minerals into more bioavailable forms or produce metabolites that enhance absorption.

Understanding these bidirectional interactions offers opportunities for innovative nutritional strategies, including optimized mineral supplementation, functional feeds that support beneficial microbiota, and reduced environmental pollution from mineral excretion (Abdul Kari 2025). This is particularly relevant as aquaculture faces challenges such as antibiotic resistance, requiring alternative approaches to maintain fish health through nutrition and microbial management.

In this review, the fundamental structures and organs of fish immunity, adaptive mechanisms, mineral–microbiota interactions and their roles in fish nutrition and immunity, general mechanisms of mineral–microbiota interactions, the role of intestinal microbiota in regulating fish immune responses, and the effects of minerals and mineral complexes on the microbiota of aquatic organisms have been comprehensively evaluated. Despite growing interest in these topics, significant knowledge gaps remain regarding species-specific responses, optimal mineral formulations across different physiological stages, and the long-term ecological consequences of mineral excretion into aquatic environments. By critically evaluating recent advances across organizational levels, from cellular absorption mechanisms to ecosystem-scale aquaculture systems, this review aims to identify research priorities. These findings are expected to guide future experimental studies toward more sustainable and efficient aquaculture production.

### Key structures, organs, and adaptive mechanisms of fish immunity

**Fig. 1:**
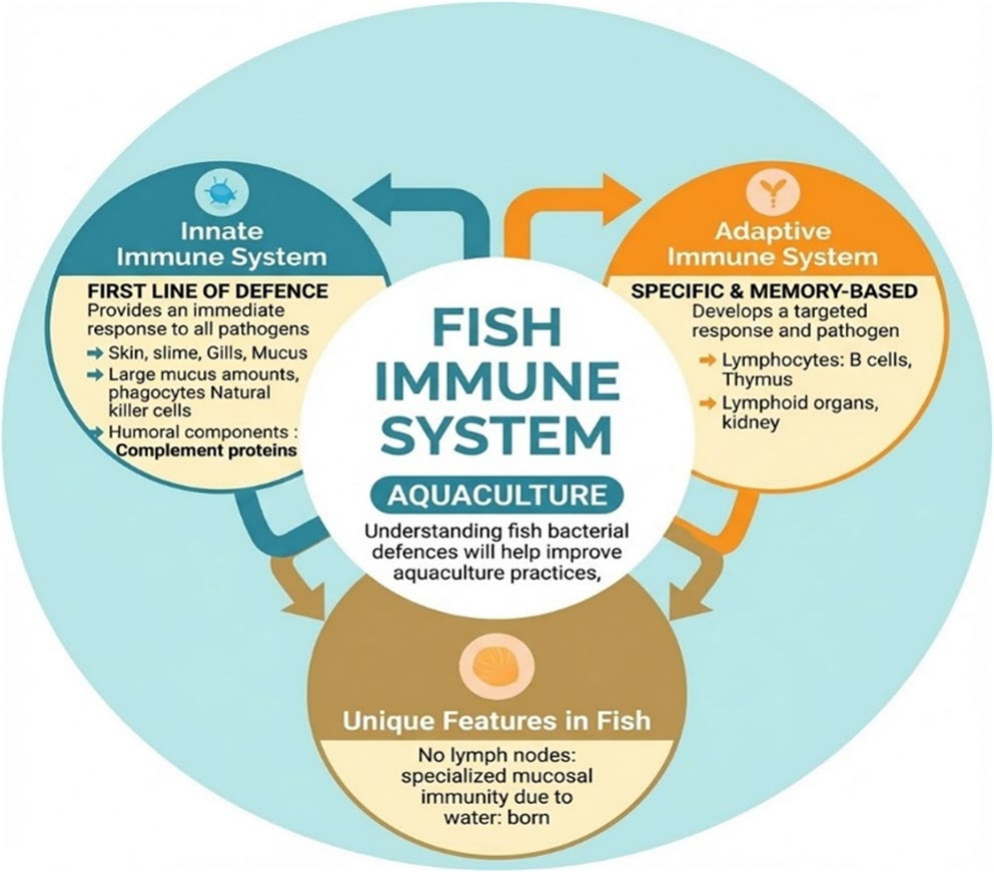
Key components of the fish immune system

Fish, as the earliest vertebrates with a backbone, provide valuable insight into the evolutionary origins of immune defense mechanisms. Their immune system consists of both innate and adaptive components, which operate synergistically to combat a broad spectrum of pathogens in aquatic environments (Fig. 1). The key components of fish immune function are illustrated in Fig. 1. The innate branch acts as the initial barrier, incorporating physical defenses such as skin and mucus, alongside cellular agents like macrophages, neutrophils, and natural killer cells. These elements respond swiftly to microbial threats and are vital for survival in microbe-rich waters (Mokhtar et al. 2023).

While less intricate than in mammals, the adaptive immune system in fish includes B and T lymphocytes, immunoglobulins, and antigen-presenting cells (Fig. 1). In teleosts, innate immunity develops earlier than adaptive responses during ontogeny (Bedekar 2022). Immunoglobulin M (IgM) serves as the primary antibody, with some species also producing IgD and IgT/IgZ, which play roles in mucosal defense. Central immune organs include the thymus, spleen, head kidney, and gut-associated lymphoid tissue (GALT) (Salinas et al. 2011; Mokhtar et al. 2023). In mammals, the regions where antigens are sampled from mucosal surfaces and where naïve T and B lymphocytes are activated are referred to as inductive sites, collectively known as mucosa-associated lymphoid tissue (MALT). According to Salinas and colleagues, MALT in teleost fish is anatomically subdivided into gut-associated lymphoid tissue (GALT), skin-associated lymphoid tissue (SALT), and gill-associated lymphoid tissue (GIALT). These tissues harbor a diverse array of leukocytes, including T cells, B cells, plasma cells, macrophages, and granulocytes. Secretory immunoglobulins, primarily produced by plasmablasts and plasma cells, play a crucial role in maintaining mucosal homeostasis. Historically, teleost B cells were believed to express only two classes of immunoglobulins IgM and IgD with IgM considered the principal antibody involved in both systemic and mucosal immune responses. However, in 2005, a third class of immunoglobulin, IgT (also referred to as IgZ in some species), was identified. Recent studies have demonstrated that IgT/IgZ functions as the dominant immunoglobulin in gut-associated mucosal immunity in teleosts (Salinas et al. 2011). The anterior kidney, in particular, functions as a major hematopoietic and immune site, effectively replacing the bone marrow found in higher vertebrates.

Fish immunity is shaped by their aquatic habitat. Constant exposure of mucosal surfaces such as skin, gills, and gut to waterborne pathogens necessitates strong localized immune responses (Fig. 1). To meet this challenge, fish have developed specialized mucosal antibodies and antimicrobial peptides. Environmental variables like temperature, salinity, and pollution further modulate immune activity, contributing to a highly responsive and adaptable system. The maturation timeline of the adaptive immune system varies across species (Bedekar 2022), and although innate defenses are broad and diverse, adaptive immunity becomes especially important when stimulated by vaccination. Its efficacy is influenced by factors such as age, thermal conditions, physiological stress, and the route of vaccine delivery.

The innate immune responses of various commercially important fish species were comprehensively reviewed by Whyte (2007), who emphasized that understanding the protective mechanisms of the immune system is crucial when considering alternative species to traditional Salmonidae for sustainable aquaculture. This knowledge is essential for effectively managing disease outbreaks and implementing preventive strategies to enhance disease resistance in aquaculture environments. Studying fish immune systems not only deepens our understanding of vertebrate immunology but also informs practical approaches in aquaculture, conservation, and disease management. Recent research continues to uncover the complexity and adaptability of immune responses in these aquatic vertebrates, highlighting their capacity for sophisticated defense (Mokhtar et al. 2023). Building on this foundation, Zhang et al. (2024) conducted a comprehensive review of the interactions between the immune system and intestinal microbiota in fish, summarizing how the host immune system shapes the microbial community and, conversely, how the microbiota regulates immune responses. Complementing this, Auclert et al. (2024) analyzed the intertwined processes of immune maturation and microbial community succession during fish development, discussing the patterns that support a healthy transition to the adult stage. More recently, Sharifinia (2025) demonstrated that the integration of chelated minerals into aquaculture diets enhances nutrient bioavailability, immune function, and disease resistance in both fish and shrimp, thereby supporting more sustainable aquaculture production. These minerals were shown to improve feed efficiency, growth performance, and resilience to stress, while also reducing heavy metal toxicity and oxidative stress. The authors emphasized that further research is needed to clarify the underlying mechanisms, species-specific responses, and potential ecological impacts. With the development of evidence-based guidelines and interdisciplinary collaboration, chelated minerals hold considerable potential to advance aquaculture practices and improve the welfare of aquatic animals.

### General mechanisms of mineral microbiota interactions in fish

The mechanism of interaction between minerals and the fish microbiota is unique and complex, as it varies depending on both the aquatic environment in which the fish live and their feeding habits (whether carnivorous, omnivorous, or herbivorous). Marine fish obtain most of their minerals directly from the water through osmoregulation, whereas freshwater fish primarily acquire minerals from the food they consume. All fish perform osmoregulation. Since mineral concentrations are higher in open seas compared to freshwater environments, this difference is significant (Mmanda 2025). The intestinal microbiota regulates the activity of enzymes such as Na⁺/K⁺-ATPase, which maintain ionic balance in the fish’s body, thereby optimizing mineral absorption according to the salinity of the environment. Because fish both rely on osmoregulation and differ in their feeding strategies, species-specific and mineral-specific studies are essential (each fish species should be studied in relation to each mineral). Based on current research, the mechanism can be explained in three stages: First stage enzymatic breakdown: In aquaculture, plant-based feeds such as soybean, corn, and wheat contain minerals like phosphorus, zinc, and magnesium bound to phytic acid molecules (Mmanda 2025). Many fish cannot produce the phytase enzyme required to break down this acid. However, intestinal bacteria such as *Bacillus* and *Lactobacillus* can secrete phytase, breaking the chemical bonds and releasing minerals into a form that can be absorbed (Das and Ghosh 2013). This researchers have demonstrated that symbiotic bacteria such as *Bacillus subtilis*, which live in the intestines of freshwater fish, produce the enzyme phytase and thereby break down phytate that traps minerals in plant-based feeds. The same researchers, in addition to the possible use of exogenous phytase, have suggested the direct inclusion of organisms like *B. subtilis* as probiotics in diets, emphasizing that this approach could enhance the availability of essential minerals and nutrients in plant-based diets.

Second stage chemical modification of the environment (pH balance): For minerals to be absorbed, the intestinal environment must maintain a certain level of acidity. Beneficial bacteria ferment carbohydrates in the diet, producing short-chain fatty acids and lactic acid. These acids lower the pH in the intestinal lumen, increasing the solubility of calcium and magnesium, and facilitating their passage into the bloodstream through microvilli (Ringø et al. 2010; Wee et al. 2024).

Third stage activation of transport genes and cells: Bacteria not only release minerals but also signal intestinal cells to increase mineral uptake when absorption is insufficient. They enhance the genetic expression of mineral transport proteins in epithelial cells for example, DMT1 for iron or calbindin for calcium. This increases the number of “gates” in the intestinal wall, thereby raising the amount of minerals absorbed per unit time. Importantly, each mineral follows a distinct pathway and has a specific receptor on the cell membrane. Therefore, each mineral must be studied individually. DMT1 (Nramp-β/γ) in fish has been well documented in the absorption of iron and other divalent metals from the intestine. Calbindin-D28K, on the other hand, has been reported mainly in the nervous system and in branchial neuroendocrine cells of fish; its role in intestinal calcium absorption is not as well elucidated as in mammals/birds. Calbindin-D9K is absent in fish, as it is specific to mammals only (Kwong 2024).

### Mineral–microbiota interactions and their roles in fish nutrition and immunity

Nutrition plays a pivotal role in modulating fish growth, metabolism, and resilience to environmental stressors, thereby providing a non-pharmaceutical strategy to enhance immune responses and disease resistance in aquaculture species (Dawood et al. 2022; Abdul Kari 2025). This field of investigation, termed nutritional immunity, encompasses the influence of dietary components on fish immune function and the augmentation of both innate and adaptive immune responses (Mendivil 2021; Qi et al. 2024). Among the essential nutrients that perform specific functions in immune pathways are vitamins, minerals, and amino acids (Shastak and Pelletier 2024; Bagheri et al. 2024; Abdul Kari 2025; Salamanca et al. 2025).

Of these essential nutrients, minerals constitute a fundamental component of fish nutrition and immune function, exerting considerable influence on growth, metabolism, disease resistance, and overall health. The balance and bioavailability of these micronutrients are essential prerequisites for sustainable aquaculture practices and optimal fish performance. Key minerals, including zinc, selenium, and iron, serve critical functions as cofactors in enzymatic reactions within immune cells and as structural constituents of immune-related proteins (Lall and Kaushik 2021; Lall 2022). These inorganic elements, required in relatively small quantities for various physiological processes, are conventionally categorized into two groups: macrominerals, which are needed in larger amounts (e.g., calcium, phosphorus, magnesium, sodium, potassium), and microminerals or trace elements, which are required in minute quantities (e.g., iron, zinc, copper, selenium, iodine). Fish acquire minerals through two primary routes: dietary intake and direct absorption from the aquatic environment. Unlike terrestrial animals, fish possess the unique capability to absorb certain minerals directly through their gills and integument from the surrounding water (Lall and Kaushik 2021).

Given their diverse sources and forms, minerals perform a wide array of physiological functions that are indispensable for fish health and development. Calcium and phosphorus are particularly critical for bone formation and the maintenance of skeletal structural integrity. Electrolyte minerals such as sodium, potassium, and chloride are essential for maintaining fluid balance and ionic regulation, processes that are fundamental to cellular homeostasis. Additionally, magnesium, zinc, and copper function as cofactors in numerous metabolic enzymes, facilitating essential biochemical reactions. Adequate mineral intake supports proper tissue development and reproductive success in fish populations. Conversely, mineral deficiencies or imbalances can result in compromised growth, skeletal deformities, reduced feed conversion efficiency, and elevated mortality rates (Lall and Kaushik 2021; Niyompano 2024).

In addition to these fundamental physiological functions, minerals exert significant immunomodulatory effects in fish. Zinc and selenium enhance antioxidant defense mechanisms and support leukocyte function, thereby strengthening cellular immunity. Iron is indispensable for hemoglobin synthesis and plays a crucial role in pathogen resistance. Copper contributes to the activity of enzymes involved in immune signaling pathways, while iodine supports thyroid function, which indirectly influences immune competence and stress resilience. The concept of nutritional immunomodulation utilizing minerals and other nutrients to enhance immune function has gained increasing recognition as a sustainable strategy in aquaculture to minimize antibiotic dependence and improve disease resistance (Abdul Kari 2025).

Translating these nutritional and immunological insights into practice, the application of mineral nutrition in aquaculture systems requires careful consideration of multiple factors. The bioavailability and absorption efficiency of minerals are influenced by dietary composition, water quality parameters, and species-specific physiological characteristics. To meet the diverse nutritional requirements of different fish species, mineral premixes or fortified feeds are commonly employed in commercial aquaculture operations. However, excessive mineral discharge from aquaculture facilities can adversely impact aquatic ecosystems, underscoring the importance of precision nutrition strategies that optimize mineral utilization while minimizing environmental impact. This balanced approach ensures both the sustainability of aquaculture production and the protection of surrounding water bodies.

### The role of gut microbiota in modulating fish immune responses

**Fig. 2:**
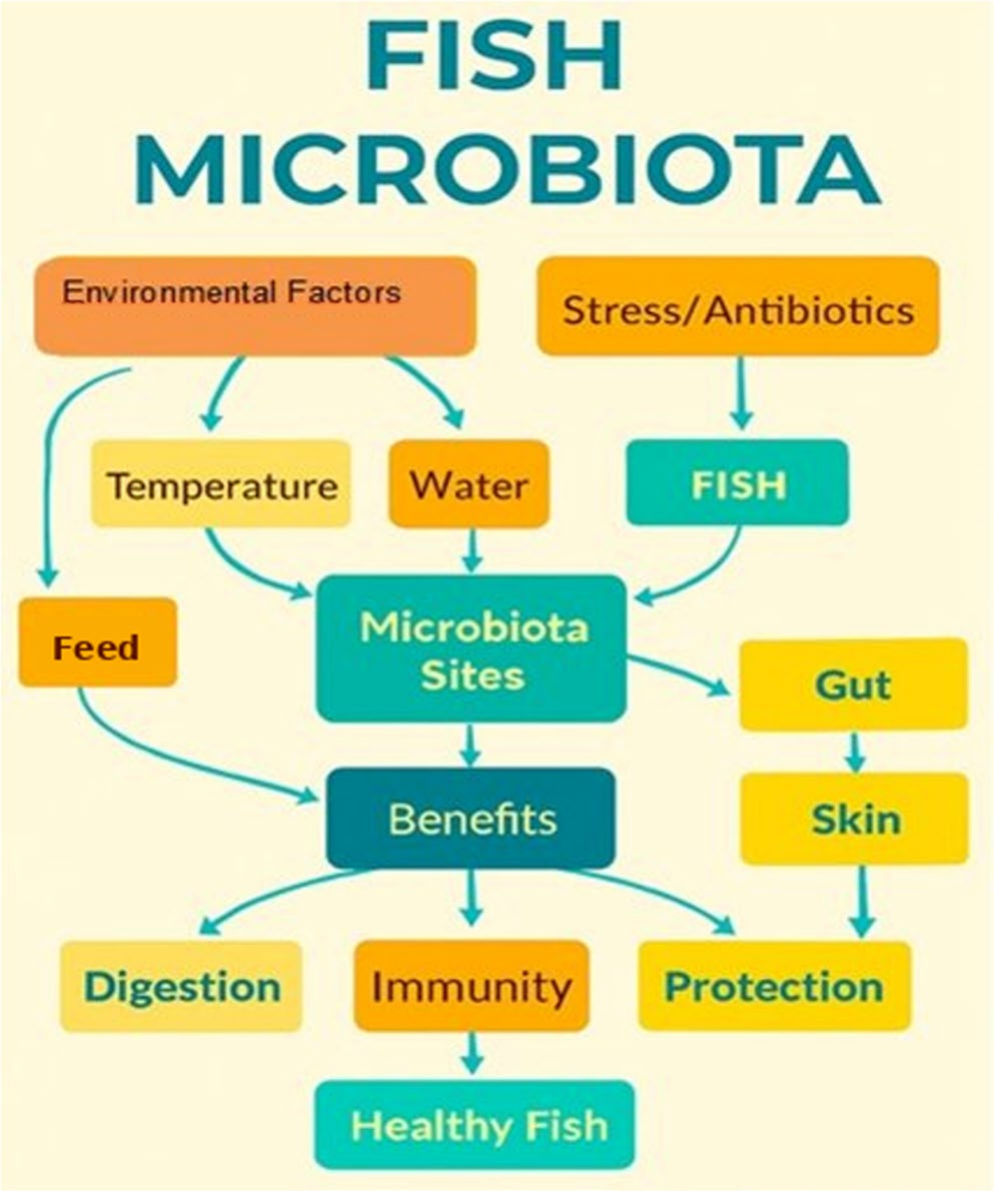
Overview of factors influencing fish microbiota and their associated health benefits

The gastrointestinal tract of fish harbors a diverse and dynamic community of microorganisms collectively known as the gut microbiota, which plays a pivotal role in host physiology by influencing digestion, nutrient absorption, immune function, and overall health (Kanika et al. 2025). Unlike terrestrial animals, fish inhabit a wide range of aquatic environments freshwater, brackish, and marine each exerting unique pressures on microbial colonization and composition. Factors such as diet, habitat, developmental stage, and host genetics shape the structure and function of the gut microbiota, resulting in considerable interspecies variability (Nawaz et al. 2018; Moroni et al. 2025). Recent advances in high-throughput sequencing and metagenomics have illuminated the complexity of these microbial ecosystems, revealing not only their taxonomic diversity but also their functional potential (Thormar et al. 2024). The multifactorial regulation of fish microbiota encompassing environmental conditions such as temperature and water quality, dietary mineral inputs, and physiological stressors including antibiotic exposure is illustrated in Fig. 2.

Critically, the gut microbiota does not operate independently of the nutritional environment. As detailed in preceding sections, dietary minerals including iron, zinc, magnesium, selenium, manganese, and copper exert significant selective pressure on microbial community composition. For instance, organic and nanoparticle mineral forms have been shown to enrich beneficial taxa such as *Lactobacillus*, *Lactococcus*, and *Weissella* while reducing pathogenic genera including *Vibrio* and *Aeromonas* across multiple aquaculture species (Yang et al. 2025; Silva et al. 2024; Xu et al. 2021). Conversely, the microbiota itself regulates mineral bioavailability through phytase secretion, pH modulation via short-chain fatty acid production, and upregulation of intestinal transport proteins such as DMT1 (Das and Ghosh 2013; Ringø et al. 2010; Kwong 2024). This bidirectional mineral microbiota axis therefore constitutes a functional interface between nutrition and immunity that is central to fish health.

The immune relevance of this interface is substantial. Commensal microbes stimulated or enriched by appropriate mineral supplementation contribute to the production of antimicrobial peptides, reinforce mucosal barrier integrity through goblet cell proliferation and mucus-related gene expression, and regulate inflammatory responses at the epithelial surface. Studies in zebrafish (*Danio rerio*) and large yellow croaker (*Larimichthys crocea*) have demonstrated that commensal bacteria stimulate innate immune pathways and enhance resistance to pathogenic invasion (Zhang et al. 2024; Wang et al. 2025). These effects are directly paralleled by mineral supplementation findings reviewed here: zinc supplementation at optimal doses enhanced disease resistance and beneficial midgut microbiota abundance in grouper (Wei et al. 2021), while selenium nanoparticles increased beneficial microbial populations and reduced pathogens in Nile tilapia (Zahran et al. 2025), and magnesium supplementation promoted villus length and goblet cell number in largemouth bass (He et al. 2025) structural changes that directly support mucosal immune competence.

Reciprocally, the host immune system shapes microbial composition through mucus secretion, immunoglobulin production, and epithelial signaling, while mineral status influences the capacity of these immune effectors. Iron deficiency, for example, impairs neutrophil and macrophage activity, while zinc is essential for lymphocyte development and cytokine regulation. When mineral–microbiota interactions are disrupted whether through suboptimal doses, antagonistic inter-element competition, or the use of inappropriate mineral forms both microbial homeostasis and immune competence are compromised simultaneously. The competitive interactions between Fe and Zn at shared intestinal transporters, or between long term Zn intake and Cu absorption, illustrate how mineral imbalances can cascade into microbiota dysbiosis and, consequently, immunosuppression.

Microbiota colonize specific sites, including the gut and skin, where they contribute to key host functions such as digestion, immune modulation, and pathogen protection (Zhang et al. 2024), as depicted in Fig. 2. The mineral composition of the diet is a primary determinant of whether these contributions are beneficial or detrimental, and understanding fish gut microbiota is increasingly recognized as essential for aquaculture sustainability, disease prevention, and ecological monitoring (Llewellyn et al. 2014; Kanika et al. 2025). Collectively, these interactions support the development and maintenance of healthy fish (Fig. 2), positioning mineral nutrition as a key lever through which gut microbiota and the immunity it supports can be deliberately managed in aquaculture systems.

### Mineral microbiota interactions in fish and application in aquaculture practice

Minerals play a vital role in fish physiology, influencing growth, metabolism, osmoregulation, and immune function. Recent advances in fish microbiome research have revealed that these essential nutrients also interact closely with the gut microbiota, shaping microbial composition and activity. Conversely, resident microbes can modulate mineral bioavailability and absorption, forming a bidirectional relationship that impacts host health. In aquatic environments, factors such as water mineral content, diet formulation, and microbial colonization patterns contribute to this dynamic interplay. Understanding mineral-microbiota interactions in fish is crucial for optimizing aquaculture nutrition strategies, enhancing disease resistance, and promoting sustainable fish farming practices (Luan et al. 2023; Mmanda 2025).

The intricate relationship between minerals and the microbiota in fish represents a fascinating frontier in aquatic biology with profound implications for sustainable aquaculture. As global demand for seafood continues to rise and wild fish stocks face mounting pressure, optimizing fish health and production efficiency in aquaculture systems has become increasingly critical. At the intersection of nutrition, microbiology, and fish physiology lies a complex network of interactions where dietary and environmental minerals shape the composition and function of microbial communities, while these microorganisms, in turn, influence mineral bioavailability, metabolism, and utilization in their piscine hosts (Zheng et al. 2024).

The fish microbiota comprising bacteria, archaea, fungi, and viruses that colonize the skin, gills, and gastrointestinal tract functions as a dynamic ecosystem that plays essential roles in nutrient processing, immune system development, pathogen resistance, and overall host health. Minerals such as iron, zinc, copper, selenium, and calcium serve not only as vital nutrients for fish growth and physiological functions but also as key modulators of microbial community structure and activity. These elements can influence microbial gene expression, enzyme function, and competitive dynamics among different microbial species, ultimately affecting the stability and beneficial functions of the microbiota (Kanika et al. 2025; Huang et al. 2025).

Understanding how minerals and microbiota interact in fish is no longer merely an academic pursuit but a practical necessity for advancing aquaculture practices. Mineral supplementation strategies, water quality management, and dietary formulations all have the potential to manipulate the fish microbiome in ways that enhance growth performance, disease resistance, and feed conversion efficiency (Kanika et al. 2025; Tay et al. 2025a, b). Conversely, dysbiosis an imbalance in microbial communities, can impair mineral absorption and utilization, leading to deficiencies and compromised fish health even when dietary mineral levels appear adequate (Xavier et al. 2023). In the review by Kanika and colleagues, the diversity, functions, and applications of fish gut microbiota in aquaculture were addressed. The authors emphasized that microbiota plays a critical role in nutrient digestion, immune regulation, and disease resistance, and also highlighted that microbiota management, in terms of biological protection, can support the health and sustainability of species (Kanika et al. 2025). Tay et al. (2025a, b) discussed the effects of gut microbiota on the overall health of fish raised in aquaculture. They linked microbiota balance to growth performance, feed efficiency, and the strengthening of the immune system, and drew attention to the potential of probiotic and prebiotic applications to improve fish health. The current state of research on fish microbiota was summarized by Luan et al. (2023). It was noted that microbiota studies have important applications in enhancing aquaculture productivity, disease control, and resilience against environmental stress factors, and it was further emphasized that microbiota-based nutritional strategies should be developed in the future.

This exploration of mineral-microbiota interactions in fish aims to bridge fundamental scientific knowledge with practical aquaculture applications, examining how these relationships can be harnessed to develop innovative strategies for sustainable fish production, reduced antibiotic use, and improved fish welfare in an industry that feeds billions worldwide.

In the following section, the studies summarized in Table 1 will be examined one by one in sequential order to provide a clearer understanding of their methodologies and outcomes.

**Table 1:** Mineral effects on intestinal function of aquatic species

Aquaculture species	Fish weight (g)	Trace mineral type	Inclusion level	Admin period	Best dose/doses	Effect	References
Yellow catfish (*Pelteobagrus fulvidraco*)	2.25 ± 0.00	Fe_2_O_3_ NPs (iron oxide nanoparticles)	22.23, 28.28, 36.10, 44.53, 52.21, and 61.85 mg Fe/kg diet	10 weeks	44.53 mg Fe/kg	Enhanced intestinal histological features	Wei et al. 2025
Largemouth bass (*Micropterus salmoides*)	4.11 ± 0.09	Fe (Iron)	42.26, 50.79, 66.61, 80.86, 123.13, and 201.87 Fe Fe mg/kg	10 weeks	80.86 mg/kg and 201.87 Fe mg/kg	intestinal flora showed that the such as *Plesiomonas*,* Peptostreptococcaceae*, and *Lactococcus* in the genus level increased	Mao et al. 2024
Golden pompanos (*Trachinotus ovatus*)		ZnSO_4_ (Zinc sulphate) and nZnO (nano-Zn)	60 mg Zn kg − 1 ZnSO_4_ 60 mg Zn kg − 1 nZnO or 170 mg Zn kg − 1 nZnO	5 weeks	60 mg Zn kg−	Enhanced probiotic colonization (lactic-acid *bacilli* spp.) in the intestine. Increased mucus-related gene expression and enhanced goblet cell renewal in villi.	Ma and Wang 2023
Pearl gentian grouper (*Epinephelus lanceolatus*♂×*Epinephelus fuscoguttatu*s♀)	12.00 ± 0.01	ZnSO_4_•7 H_2_O (Zinc sulfate)	20, 40, 60, 80, 100 and 150 mg Zn/kg	8-weeks	44.52 mg/kg	Enhanced growth, antioxidant activity, disease resistance, and midgut beneficial flora.	Wei et al. 2021
Largemouth bass (*Micropterus salmoides*)	38.24 ± 2.37	MgH_2_ (Magnesium hydride)	10 mg/kg 20 mg/kg and 30 mg/kg	8 weeks	20 mg/kg	Increased villus length, basal layer thickness, and goblet cell number. Higher *Fusobacteriota* and *Proteobacteria*, lower *Firmicutes* compared to control.	He et al. 2025
Oriental river prawn (*Macrobrachium nipponense*)	0,151 ± 0,003	MgSO₄ (Magnesium sulfate)	1.1, 1.4, 1.6, 2.1, 2.8 and 4.5 g/kg	8 weeks	1.6 to 2.2 g kg − 1	Lower cumulative mortality and higher antioxidant capacity under ammonia stress and supports growth and well-being. Epithelial cell detachment and alteration at the lowest/highest Mg levels.	Kong et al. 2022
Chinese tongue sole (*Cynoglossus semilaevis*)	120.45 ± 0.45	Se (Nano-selenium)	1.6 mg/kg, and 2.4 mg/kg	60 days	1.6 mg/kg, and 2.4 mg/kg	Low overall diversity with Vibrio > 90% across all groups; low- and high-selenium groups showed distinct shifts with enriched *Acinetobacter*, *Arthrobacter*, and *Streptophytes*	Jia et al. 2022
Nile tilapia (*Oreochromis niloticus***)**	57.60 ± 0.56	SeNPs (Selenium nanoparticles)	0.75 and 1.5 mg/kg	8 weeks	1.5 mg/kg	Increased beneficial microbes and reduced pathogens, indicating improved growth and intestinal function.	Zahran et al. 2025
Yellow catfish (*Pelteobagrus fulvidraco*)	2.69 ± 0.01	MnSO_4_⋅H_2_O (Mn sulfate monohydrate), MnO_2_ (Mn dioxide), MnO_2_NPs (Mn dioxide nanoparticles), Mn-Gly (Mn glycine chelate), Mn-MHA (hydroxymethionine-chelated Mn)	0.008 g/kg 0.003 g/kg 0.004 g/kg 0.01 g/kg 0.022 g/kg	10 weeks.	Mn-Gly 0.01 g/kg Mn-MHA 0.022 g/kg	Organic supplements (Mn-Gly and Mn-MHA) improved growth, feed utilization, and intestinal health.	Xu et al. 2023
Pacific white shrimp (*Litopenaeus vannamei*)	0.95 ± 0.01	MnSO_4_.H_2_O (Manganese sulfate)	10.04, 27.18, 39.57, 54.57, 72.67 and 111.97 mg/kg,	8 weeks	39.57 and 111.97 mg/kg	Mn supplementation markedly altered intestinal microbiota composition and function.	Jiao et al. 2021
White shrimp (*Penaeus vannamei*)	5.29 ± 0.03	CuSO_4_·5H_2_O (Inorganic copper), Cu-proteinate (Organic copper)	30 mg/kg	8 weeks	Organic copper 30 mg/kg	Organic copper modulated microecological niches and enhanced microbiota stability and functional abundance in the intestine, gill, and water.	Yang et al. 2025
Pacific white shrimp (*Litopenaeus vannamei*).	1.86 ± 0.03	no-supplemented copper, CuSO4·5H2O (Copper sulfate), CuSO4·5H2O+Availa^®^Cu100 (copper amino acid complex) and Availa^®^Cu100)	10.23, 40.64, 39.70 and 25.07 mg Cu/kg diet	8 weeks	-	Copper sources did not affect intestinal bacterial richness or diversity. Bacteroidetes abundance: Diet M (21.11%), Diet Availa (27.15%). *Vibrionaceae* decreased in Diet M and Availa vs. Diet NSC and CS. *Flavobacteriaceae* and *Alteromonadaceae* increased in Diet CS, M, and Availa. *Mycoplasmataceae* decreased in these diets. No significant differences among treatments.	Yuan et al. 2019

In a study by Wei et al. (2025), yellow catfish (*Pelteobagrus fulvidraco*; initial weight ~ 2.25 g) were fed diets supplemented with iron oxide nanoparticles (Fe₂O₃ NPs) at six graded levels (22.23–61.85 mg Fe/kg) for 10 weeks. The optimal dose of 44.53 mg Fe/kg was found to enhance intestinal histological features. Recording the optimal dose in this study is highly important. However, since this fish species is omnivorous, the experiment should be repeated in carnivorous and herbivorous fish species. Another study investigating the effects of iron supplementation on the intestinal microbiota of carnivorous largemouth bass (*Micropterus salmoides*) was conducted by Mao et al. (2024). Mao et al. (2024) examined dietary iron supplementation in largemouth bass (~ 4.11 g) across six levels (42.26–201.87 mg Fe/kg) over 10 weeks. At doses of 80.86 and 201.87 mg Fe/kg, shifts in intestinal microbiota were observed, including increased abundance of genera such as *Plesiomonas*, *Peptostreptococcaceae*, and *Lactococcus* (Table 1).

In both studies, feeding trials lasted only 10 weeks. In aquaculture, 10 weeks is a relatively short period. For example, the long-term effects of iron supplementation over one year remain unknown. Results may also vary across different sizes and age groups, and the interactions of iron with other trace elements have not been investigated. The effects of zinc on the intestinal microbiota of golden pompano (*Trachinotus ovatus*) were studied by Ma and Wang (2023). They compared zinc sulfate (ZnSO₄) and nano-zinc oxide (nZnO) supplementation in golden pompano at 60 mg Zn/kg (ZnSO₄), 60 mg Zn/kg (nZnO), and 170 mg Zn/kg (nZnO) for 5 weeks. The optimal dose of 60 mg Zn/kg enhanced probiotic colonization of lactic acid bacteria in the intestine and increased mucus-related gene expression and goblet cell renewal in villi. Although the duration of this study was short, the inclusion of genetic and molecular analyses was significant.

Wei et al. (2021) supplemented pearl gentian grouper (*Epinephelus lanceolatus ♂ × E. fuscoguttatus ♀*; ~12 g) with ZnSO₄·7 H₂O at levels of 20–150 mg Zn/kg for 8 weeks. The optimal dose of 44.52 mg Zn/kg enhanced growth performance, antioxidant activity, disease resistance, and the abundance of beneficial midgut microbiota. More recently, He et al. (2025) investigated the effects of magnesium on intestinal microbiota. Largemouth bass (*Micropterus salmoides*; ~38.24 g) were fed magnesium hydride (MgH₂) at 10, 20, and 30 mg/kg for 8 weeks. The dose of 20 mg/kg increased villus length, basal layer thickness, and goblet cell number, and was associated with higher relative abundance of *Fusobacteriota* and *Proteobacteria* and lower *Firmicutes* compared to the control group. Kong et al. (2022) examined MgSO₄ supplementation in Oriental river prawn (*Macrobrachium nipponense*; ~0.151 g) at levels of 1.1–4.5 g/kg for 8 weeks. The optimal range of 1.6–2.2 g/kg reduced cumulative mortality, enhanced antioxidant capacity under ammonia stress, and supported growth and wellbeing. Epithelial cell detachment and structural alterations were noted at the lowest and highest Mg levels. The effects of selenium on intestinal microbiota were studied in Chinese tongue sole (*Cynoglossus semilaevis*) by Jia et al. (2022) and in Nile tilapia (*Oreochromis niloticus*) by Zahran et al. (2025). Jia et al. (2022) assessed nano-selenium supplementation in Chinese tongue sole (~ 120.45 g) at 1.6 and 2.4 mg/kg for 60 days. Both doses induced notable shifts in intestinal microbiota, including enrichment of *Acinetobacter*, *Arthrobacter*, and *Streptophytes*, while overall microbial diversity remained low with *Vibrio* dominating (> 90%) across all groups. Zahran et al. (2025) supplemented Nile tilapia (~ 57.60 g) with selenium nanoparticles (SeNPs) at 0.75 and 1.5 mg/kg for 8 weeks. The 1.5 mg/kg dose increased the abundance of beneficial microbes and reduced pathogenic bacteria, indicating improved growth and intestinal function. The effects of manganese on the intestinal microbiota of yellow catfish (*Pelteobagrus fulvidraco*) were investigated by Xu et al. (2023). The researchers compared five forms of manganese supplementation MnSO₄·H₂O, MnO₂, MnO₂ nanoparticles, Mn-glycine chelate (Mn-Gly), and hydroxymethionine-chelated Mn (Mn-MHA) in yellow catfish (~ 2.69 g) for 10 weeks. Organic forms (Mn-Gly at 0.01 g/kg and Mn-MHA at 0.022 g/kg) produced the most beneficial outcomes, improving growth, feed utilization, and intestinal health. Jiao et al. (2021) supplemented Pacific white shrimp (*Litopenaeus vannamei*; ~0.95 g) with MnSO₄·H₂O at six levels (10.04–111.97 mg/kg) for 8 weeks. Mn supplementation at 39.57 and 111.97 mg/kg markedly altered the composition and functional profile of the intestinal microbiota. Yang et al. (2025) compared inorganic copper (CuSO₄·5 H₂O) and organic copper (Cu-proteinate) at 30 mg/kg in white shrimp (*Penaeus vannamei*; ~5.29 g) for 8 weeks. Organic copper modulated microecological niches and enhanced microbiota stability and functional abundance in the intestine, gill, and surrounding water. Yuan et al. (2019) examined four dietary copper treatments in Pacific white shrimp (*Litopenaeus vannamei*; ~1.86 g)—no copper supplementation, CuSO₄·5 H₂O alone, CuSO₄·5 H₂O combined with an amino acid complex (Availa^®^Cu100), and Availa^®^Cu100 alone—for 8 weeks. Copper sources did not significantly affect intestinal bacterial richness or diversity overall; however, *Bacteroidetes* abundance was higher in the mixed and Availa groups (21.11% and 27.15%, respectively), *Vibrionaceae* decreased with both mixed and Availa diets, and *Flavobacteriaceae* and *Alteromonadaceae* increased in the copper sulfate, mixed, and Availa groups. *Mycoplasmataceae* abundance decreased in these same dietary groups. No statistically significant differences were found among treatments overall (Table 1).

Taken together, these studies are highly valuable in their specific contexts. However, all experiments were conducted on fish and shrimp of particular sizes and age groups, with different doses of elements and nanoparticles applied. Moreover, the maximum trial durations were 10 weeks, and even Jia et al. (2022) conducted only a 60-day experiment with microelements. Such durations are relatively short for aquaculture. If any of the above-mentioned microelements or nanoparticles are to be incorporated into commercial feeds, longer-term trials are essential. A second critical consideration is the interaction between microelements. For example, Fe and Zn share the same transport systems in the fish intestine, so high doses of Fe can suppress Zn absorption. Long-term Zn intake can reduce Cu absorption, leading to copper deficiency. Nano-Fe, Nano-Zn, Nano-Cu, and Nano-Mn particles exhibit higher bioavailability compared to conventional mineral forms. However, when used simultaneously, competitive interactions may be stronger; for instance, Nano-Fe and Nano-Zn together can cause more pronounced competition at the transporter level. Nano-particles can also influence intestinal microbiota, thereby indirectly altering the metabolism of other elements. For these reasons, before recommending their inclusion in feed formulations, the interaction levels among micro and nano elements must be clarified.

Studies on the effects of mineral complexes on the intestinal health of aquatic organisms were examined in the order presented in Table 2.

**Table 2:** Impact of mineral complexes on intestinal health in aquatic species

Aquaculture species	Fish weight (g)	Trace mineral type	Inclusion level	Admin period	Best dose/doses	Effect	References
White Shrimp (*Penaeus vannamei*)	7.21 ± 0.04	Organic and inorganic trace mineral supplementation (Zn, Cu, Mn, Fe, and Se) IM100 (inorganic), IM50 (inorganic) OM50 (organic), OM33 (organic)	standard levels of trace minerals of Zn, Cu, Mn, Fe, and Se 120 mg/kg, 30 mg/kg, 20 mg/kg, 30 mg/kg, and 0.3 mg/kg, respectively	8 weeks	Organic trace mineral supplementation	Improved gut health performance	Huang et al. 2025
Channel catfish (*Ictalurus punctatus*)	26.5 ± 0.8	Iron nanoparticles (IronNP), Copper nanoparticles (CopperNP), CopperNP + IronNP, and inorganic iron and copper (FeSO_4_ and CuSO_4_).	595.0 99.3 482.0 + 94.7 637.0 + 25.9	9 weeks	Cu nanoparticles	Cu nanoparticles increased the relative abundance of lactic acid taxa (e.g., *Weissella*, Leuconostocaceae, Lactobacillales, Streptococcaceae, *Lactococcus*, Actinobacteriota),	Silva et al. 2024
Nile tilapia (*Oreochromis niloticus*)	91.75–95.75	Metal-amino acid complexes, MAAC (Zn, Se, Cu, Fe and Mn)	0, 25%, 50%, 75% and 100% MAAC	80 days	about 50% to 55% substitution	Optimum growth performance, feed digestion, absorption and efficiency, beneficial gut microbiota	El-Sayed et al. 2023
Zebrafish (*Danio rerio*)	0.26 ± 0.03	Organomineral chelated trace element compounds (Fe (iron), Mn (manganese), Zn (zinc), Se (selenium), I (iodine), Cu (copper))	Fe 10, Mn 15, Zn 35, Se 0.3, I 1.1, Cu 3 g/L	60 days	Chelated micronutrients	Altered microbiota in anterior and posterior intestine	Nikiforov-Nikishin et al. 2022
Largemouth bass (*Micropterus salmoides*)	7.96 ± 0.19	Azomite (natural trace mineral complex)	1.0, 2.0, 3.0, 4.0, 5.0 and 6.0 g/kg	60 days	High Azomite levels Azomite-treated groups	High Azomite levels may negatively affect beneficial bacteria and disrupt microflora balance *Aeromonas* levels in the gut were lower in Azomite-treated groups.	Xu et al. 2021

To combine the cost advantage of inorganic forms with the high bioavailability of organic forms, Huang et al. (2025) investigated the effects of organic and inorganic trace mineral supplementation in white shrimp (Penaeus vannamei; ~7.21 g) over 8 weeks. Four dietary treatments were tested: 100% inorganic minerals (IM100), 50% inorganic minerals (IM50), 50% organic minerals (OM50), and 33% organic minerals (OM33), with standard inclusion levels of Zn (120 mg/kg), Cu (30 mg/kg), Mn (20 mg/kg), Fe (30 mg/kg), and Se (0.3 mg/kg). Organic trace mineral supplementation produced the best outcomes, improving overall gut health performance compared to inorganic sources. The effects of iron and copper nanoparticles on the intestinal microbiota of channel catfish were investigated by Silva et al. (2024). The authors evaluated the effects of iron nanoparticles (IronNP), copper nanoparticles (CopperNP), a combination of CopperNP and IronNP, and conventional inorganic sources (FeSO₄ and CuSO₄) in channel catfish (*Ictalurus punctatus*; ~26.5 g) for 9 weeks. Inclusion levels were 595.0 mg/kg for IronNP, 99.3 mg/kg for CopperNP, 482.0 + 94.7 mg/kg for the combined nanoparticle group, and 637.0 + 25.9 mg/kg for inorganic iron and copper. Among all treatments, copper nanoparticles yielded the most notable intestinal effect, increasing the relative abundance of lactic acid-associated taxa including *Weissella*,* Lactococcus*, Leuconostocaceae, Streptococcaceae, Lactobacillales, and Actinobacteriota. El-Sayed et al. (2023) examined the impact of metal–amino acid complexes (MAAC) containing Zn, Se, Cu, Fe, and Mn in Nile tilapia (*Oreochromis niloticus*; 91.75–95.75 g) over 80 days. Dietary treatments consisted of 0%, 25%, 50%, 75%, and 100% MAAC substitution. Partial substitution at approximately 50–55% MAAC was identified as the optimal level, yielding the best growth performance, feed digestion, absorption and efficiency, and promoting a beneficial gut microbiota composition. Nikiforov-Nikishin et al. (2022) assessed the effects of organomineral chelated trace element compounds — including Fe (10 g/L), Mn (15 g/L), Zn (35 g/L), Se (0.3 g/L), I (1.1 g/L), and Cu (3 g/L) in zebrafish (*Danio rerio*; ~0.26 g) over 60 days. Chelated micronutrient supplementation resulted in notable alterations in the microbial communities of both the anterior and posterior intestine compared to control groups. Xu et al. (2021) evaluated the effects of Azomite, a natural trace mineral complex, in largemouth bass (*Micropterus salmoides*; ~7.96 g) at graded dietary levels of 1.0, 2.0, 3.0, 4.0, 5.0, and 6.0 g/kg over 60 days. While Azomite-treated groups demonstrated reduced *Aeromonas* levels in the gut, higher Azomite inclusion levels were associated with potential negative effects on beneficial bacterial populations and disruption of overall intestinal microflora balance. Çiçek and Özoğul (2021) reviewed studies on the effects of SeNPs on growth performance, hematological and serum biochemical parameters, and antioxidant status stimulation in fish in the presence or absence of different stress conditions. The authors reported that the potential effects of SeNPs on growth, blood, serum parameters, and antioxidant status in fish vary depending on concentration, SeNP size, application time, and fish species, and recommended that further studies are needed.

When the studies are examined as a whole, several important limitations become apparent. First, the existing research has focused on only a limited number of species, including channel catfish, Nile tilapia, zebrafish, largemouth bass, and white shrimp, while coldwater fish such as trout and salmon, marine fish species, and other commercially important taxa remain understudied. Second, experimental periods were relatively short, ranging from 8 to 80 days (Tables 1 and 2), and none encompassed a complete reproductive cycle, leaving long-term exposure effects, chronic toxicity, and transgenerational impacts (F1 and F2 generations) unresolved. Third, the mechanisms by which nanoparticles and organic/inorganic mineral forms influence intestinal microbiota have not been fully elucidated, optimal doses specific to species, age, and physiological status have not been established, and the high IronNP dose used by Silva et al. (2024) (595 mg/kg) raises potential toxicity concerns. Furthermore, synergistic or antagonistic interactions among Zn, Cu, Mn, Fe, and Se remain inadequately characterized, and comparative evaluations of combined mineral formulations are limited, as single-mineral studies predominate. Although Çiçek and Özoğul (2021) highlighted the importance of stress conditions, most studies were conducted under controlled laboratory settings without simulating real aquaculture stressors such as stocking density stress or water quality fluctuations. Finally, the ecotoxicological consequences of nanoparticles entering aquatic environments, their bioaccumulation potential, and mineral residue levels in fish tissues from a food safety perspective have not been sufficiently investigated.

To address these gaps and ensure the long-term sustainability of aquaculture, future research should broaden species diversity and extend experimental designs to cover full reproductive cycles, given that chronic toxicity and epigenetic effects transmitted across generations warrant thorough investigation. Clarifying the intestinal absorption mechanisms, tissue distribution, and excretion pathways of nanoparticles and organic mineral complexes is essential, and isotope tracing techniques could prove valuable in tracking the fate of minerals within the body. The functional capacity of the intestinal microbiota should be assessed through metagenomic and metabolomic analyses, complemented by transcriptomic and proteomic approaches to investigate gene expression-level responses. Comparative trials that simultaneously evaluate nanoparticle, organic, and inorganic mineral forms within the same experimental design are needed, and the potential synergistic effects of combining SeNPs with organic mineral complexes merit further exploration. Laboratory findings should be validated under commercial farm conditions encompassing varying stocking densities, temperatures, and water quality parameters, and the ecotoxicological effects of nanoparticles released into aquatic environments should be systematically modeled and measured. Finally, a comprehensive cost-benefit analysis of organic, inorganic, and nanoparticle mineral sources is necessary to evaluate their commercial viability in aquaculture production.

## Discussion

The studies reviewed in this paper collectively underscore the pivotal and bidirectional nature of mineral–microbiota interactions in farmed aquatic species, yet they also expose a series of recurrent methodological constraints that limit the generalizability of current findings. A synthesis of the available evidence reveals several overarching themes that merit systematic discussion: the comparative efficacy of mineral forms, the ecological significance of dose optimization, the mechanistic basis of mineral–microbiota crosstalk, inter-mineral competitive dynamics, and the translational gap between controlled laboratory experiments and commercial aquaculture practice.

### Mineral form, dosage, and optimal supplementation levels in microbiota modulation

One of the most consistent patterns emerging from the reviewed literature is that organic and nanoparticle mineral forms exert more favorable and selective effects on intestinal microbiota than their conventional inorganic counterparts. In golden pompano, nano-zinc oxide (nZnO) at 60 mg Zn/kg promoted lactic acid bacteria colonization and enhanced mucus-related gene expression to a greater degree than equivalent doses of zinc sulfate (Ma and Wang 2023). Similarly, in yellow catfish, organic manganese chelates (Mn-Gly and Mn-MHA) outperformed inorganic MnSO₄·H₂O and MnO₂ in improving growth, feed utilization, and intestinal morphology (Xu et al. 2023). In white shrimp, organic copper (Cu-proteinate) modulated microecological niches and enhanced microbiota stability in the intestine, gill, and surrounding water compared to inorganic copper sulfate (Yang et al. 2025). Taken together, these findings suggest that the chemical speciation of a mineral is not merely a pharmacokinetic variable but a key ecological determinant of which microbial taxa are selectively enriched or suppressed within the gut ecosystem.

The superiority of nanoparticle forms appears to be linked to their smaller particle size, increased surface-area-to-volume ratio, and enhanced solubility, which facilitate more intimate contact with intestinal epithelial cells and microbial communities (Mmanda 2025). In channel catfish, copper nanoparticles (CopperNP) increased the relative abundance of lactic acid-associated taxa including *Weissella*,* Lactococcus*, Leuconostocaceae, and Lactobacillales whereas conventional inorganic iron and copper sources produced comparatively modest microbiota shifts (Silva et al. 2024). This pattern is further corroborated by the work of Huang et al. (2025), who demonstrated that organic trace mineral supplementation in white shrimp consistently outperformed inorganic formulations across a suite of gut health parameters, including intestinal morphology, microbial diversity, and immune-related gene expression. However, the ecological and toxicological implications of nanoparticle use must not be overlooked; the inclusion level of IronNP at 595 mg/kg employed by Silva et al. (2024) substantially exceeds levels used in other studies and may carry toxicity risks that are not yet fully characterized, both for the host and for the aquatic environment receiving mineral-laden effluents.

Across all minerals and species examined, the reviewed studies consistently demonstrate non-linear dose–response relationships, wherein intermediate supplementation levels yield the most beneficial outcomes and both deficiency and excess are associated with adverse effects. In yellow catfish, the optimal Fe₂O₃ nanoparticle dose of 44.53 mg Fe/kg produced superior intestinal histological outcomes compared to both lower and higher inclusion levels (Wei et al. 2025). For zinc in pearl gentian grouper, 44.52 mg Zn/kg was identified as the threshold dose for maximizing growth, antioxidant activity, disease resistance, and beneficial midgut microbiota abundance (Wei et al. 2021). Magnesium hydride at 20 mg/kg in largemouth bass yielded optimal improvements in villus length, basal layer thickness, and goblet cell number, whereas higher doses did not confer additional benefits (He et al. 2025). In Oriental river prawn, the optimal magnesium sulfate range of 1.6–2.2 g/kg reduced cumulative mortality and enhanced antioxidant capacity, while the lowest and highest doses were associated with epithelial cell detachment and structural intestinal alterations (Kong et al. 2022).

For combined mineral formulations, the findings of El-Sayed et al. (2023) in Nile tilapia are particularly instructive: partial substitution of inorganic minerals with metal–amino acid complexes (MAAC) at approximately 50–55% yielded optimal growth performance, feed efficiency, and beneficial gut microbiota composition, whereas complete substitution with 100% MAAC did not produce equivalent or superior results. This inverted-U dose–response pattern for combined mineral formulations suggests that a balanced integration of inorganic and organic sources may leverage complementary mechanisms of action, a hypothesis that merits further mechanistic investigation. Taken together, these dose–response data reinforce the importance of species-specific and age-specific mineral requirement studies, as the optimal dose identified for one species or life stage cannot be extrapolated to another without experimental validation.

### Mineral–microbiota axis and immune modulation: competitive dynamics and antagonistic interactions

A central theme emerging from the reviewed studies is the convergence of mineral nutrition and gut microbiota as co-regulators of fish immune function, a relationship that can be characterized as a mineral–microbiota–immunity triad. Zinc supplementation at optimal doses in pearl gentian grouper not only enhanced growth and antioxidant status but also improved disease resistance and beneficial midgut microbiota abundance, suggesting that the immune-enhancing effects of zinc are at least partially mediated through microbiota modulation (Wei et al. 2021). Similarly, selenium nanoparticles in Nile tilapia increased beneficial microbial populations while reducing pathogens, with concomitant improvements in growth and intestinal function (Zahran et al. 2025). These findings are consistent with the broader nutritional immunomodulation framework articulated by Abdul-Kari (2025) and Sharifinia (2025), which positions chelated and bioavailable mineral forms as tools for enhancing immune competence, reducing antibiotic dependence, and improving disease resistance in aquaculture.

The structural dimension of intestinal immunity is equally relevant. Magnesium supplementation at 20 mg/kg in largemouth bass increased villus length, basal layer thickness, and goblet cell number, structural parameters that are directly indicative of enhanced mucosal barrier integrity and immune competence (He et al. 2025). Goblet cell proliferation is associated with increased mucus production and secretory immunoglobulin secretion, which are frontline components of the mucosal immune response in teleosts (Salinas et al. 2011; Mokhtar et al. 2023). In contrast, the predominance of Vibrio exceeding 90% across all dietary groups in Chinese tongue sole supplemented with nano-selenium highlights the limitations of single-mineral interventions in species with inherently dysbiotic baseline microbiota profiles, and suggests that the immunological efficacy of mineral supplementation may be fundamentally constrained by the composition of the resident microbial community (Jia et al. 2022). This finding calls into question whether mineral supplementation alone is sufficient to correct deep-seated microbiota dysbiosis or whether combined strategies incorporating probiotics, prebiotics, or synbiotics are necessary to achieve meaningful immune outcomes (Aydın and Çek-Yalnız 2019).

A critical but insufficiently characterized dimension of mineral nutrition is the competitive and antagonistic interactions that occur among trace elements sharing common intestinal transport systems. Iron and zinc compete for absorption via shared divalent metal transporters, particularly DMT1 (Nramp-β/γ), such that high dietary iron supplementation can suppress zinc absorption and vice versa (Kwong 2024). Prolonged high zinc intake has been documented to reduce copper absorption in mammals, and analogous mechanisms are anticipated to operate in fish, given the shared transporter architecture. These inter-mineral competitions have direct implications for the gut microbiota: changes in luminal iron availability selectively enrich iron-scavenging pathobionts such as Vibrio and Aeromonas, whereas adequate zinc and selenium tend to favor lactic acid bacteria and Lactococcus populations (Yang et al. 2025; Zahran et al. 2025). The use of nanoparticle forms of multiple minerals simultaneously may amplify these competitive dynamics, since nano-Fe and nano-Zn particles exhibit higher bioavailability than their conventional counterparts and may therefore engage transporter competition more aggressively at lower inclusion levels (Silva et al. 2024; Mmanda 2025).

None of the single-mineral studies reviewed here systematically controlled for or measured the effect of the supplemented mineral on the intestinal concentrations of competing elements. This represents a significant methodological gap, as observed microbiota shifts attributed to a single mineral could in part reflect secondary changes in the availability of competing minerals. Multi-mineral studies such as those of Huang et al. (2025) and El-Sayed et al. (2023) are methodologically stronger in this respect because they evaluate mineral interactions implicitly through combined formulations; however, neither study included element-specific bioavailability measurements or intestinal transporter expression analyses that would allow inter-mineral competitive effects to be quantified directly. Future studies incorporating isotope dilution or stable isotope tracing techniques would substantially advance the mechanistic understanding of these competitive dynamics.

### Species- and environment-specific responses: freshwater–marine divide and long-term validity of current findings

The reviewed studies span freshwater species (yellow catfish, largemouth bass, zebrafish, Oriental river prawn, Nile tilapia), marine and estuarine species (golden pompano, grouper, channel catfish, Pacific white shrimp, Chinese tongue sole), highlighting the taxonomic diversity of aquaculture systems. However, a fundamental physiological distinction must be recognized: marine fish obtain a substantial proportion of their mineral requirements directly from the surrounding water through osmoregulation, whereas freshwater fish depend predominantly on dietary mineral intake (Mmanda 2025). The intestinal microbiota is known to regulate Na⁺/K⁺-ATPase activity, which maintains ionic homeostasis in both osmotic contexts, and the salinity of the rearing environment therefore constitutes a significant moderating variable for mineral–microbiota interactions that has not been systematically controlled across the reviewed studies. This has practical implications for aquaculture: mineral supplementation strategies validated in freshwater species may not be directly transferable to marine or brackish water systems without re-optimization of inclusion levels and mineral forms.

A further concern is the near-total absence of coldwater salmonid species (trout, salmon) and other commercially important taxa from the reviewed studies. Salmonids account for a large proportion of global aquaculture production by value and are raised under conditions (intensive recirculating aquaculture systems, cold oxygenated water, high-protein diets) that differ fundamentally from the warm-water polyculture systems in which most reviewed species were studied. The microbiota of salmonids is compositionally distinct from that of warm-water omnivores, with differences in dominant phyla and functional guilds that may result in markedly different responses to mineral supplementation (Llewellyn et al. 2014; Moroni et al. 2025). The feeding strategy of the host species also modulates mineral–microbiota interactions: carnivorous species such as grouper and largemouth bass consume high-protein, low-phytate diets, which reduces the relevance of phytase-secreting bacteria for mineral release, whereas omnivorous and herbivorous species consuming plant-based diets are more dependent on microbiota-mediated phytate hydrolysis for mineral bioavailability (Das and Ghosh 2013).

A universal limitation of the reviewed literature is the short duration of experimental trials, ranging from 5 to 80 days (Tables 1 and 2), with the majority conducted over 8–10 weeks. No study encompassed a complete reproductive cycle, meaning that the effects of mineral supplementation on reproductive performance, gonadal development, egg quality, and F1 offspring health remain entirely uncharacterized for the mineral forms and species reviewed here. Microbiota community succession in fish follows developmental stage-specific patterns that extend across months to years (Auclert et al. 2024), and it is therefore conceivable that the microbiota shifts observed in 8-week trials represent transient responses rather than stable ecological modifications. This temporal limitation is particularly critical when evaluating the safety of nanoparticle mineral forms, as chronic low-level accumulation in gut tissues, disruption of mucosal barrier integrity, and potential transgenerational epigenetic effects are phenomena that manifest over timescales far exceeding those of the reviewed experiments (Çiçek and Özoğul 2021).

Furthermore, all reviewed studies were conducted under controlled laboratory conditions that do not adequately simulate the complexity of commercial aquaculture environments, including variable stocking densities, fluctuating water temperature and quality, co-infections, and feed composition heterogeneity. The findings of Xu et al. (2021) in largemouth bass, which showed that high Azomite inclusion levels disrupted intestinal microflora balance despite reducing Aeromonas levels, illustrate the context-dependence of mineral effects and suggest that outcomes observed under optimal laboratory conditions may not reliably predict those in production settings where multiple stressors operate simultaneously. Validation of promising mineral supplementation strategies under commercial farm conditions is therefore an essential and currently missing step in the translational pipeline from bench to farm.

### Ecotoxicological sustainability and methodological gaps in future research directions

The environmental dimension of mineral supplementation in aquaculture has received insufficient attention in the reviewed literature, particularly with respect to nanoparticle forms. Nanoparticles excreted in feces or leached from feed pellets into the surrounding water column represent a novel category of environmental contaminant with unknown long-term effects on aquatic ecosystems, sediment microbial communities, and non-target organisms (Mmanda 2025; Lall and Kaushik 2021). Unlike conventional inorganic mineral salts, which dissolve predictably and are subject to well-characterized geochemical cycling, nanoparticles may persist in sediments, undergo surface transformations, and interact with natural organic matter in ways that alter their bioavailability and toxicity to wild aquatic fauna. The high IronNP inclusion level used by Silva et al. (2024) amplifies these concerns, as iron nanoparticle accumulation in aquatic sediments has been associated with shifts in sediment redox chemistry and microbial community composition in non-aquaculture contexts. A comprehensive ecotoxicological risk assessment framework, integrating both aquaculture performance data and environmental fate modeling, is urgently needed before nanoparticle mineral forms can be responsibly recommended for widespread commercial use. This gap is particularly concerning given the rapid pace at which nanoparticle-based feed additives are being developed and promoted in the aquaculture industry (Çiçek and Özoğul 2021).

The methodological landscape of the reviewed studies reveals a field that has prioritized descriptive characterization of microbiota composition over mechanistic and longitudinal analysis. Most studies employed 16 S rRNA amplicon sequencing for microbiota profiling, a technique that provides taxonomic resolution but limited functional insight. The functional capacity of mineral-responsive microbial communities, including their ability to produce short-chain fatty acids, synthesize vitamins, biotransform minerals, and regulate intestinal immune gene expression, remains essentially uncharacterized across the reviewed species and mineral combinations. Shotgun metagenomics, metatranscriptomics, and metabolomics approaches are needed to bridge this gap and to identify the metabolic pathways through which microbiota modulation by minerals translates into host physiological outcomes (Thormar et al. 2024; Kanika et al. 2025). Integrating multi-omics approaches within longitudinal experimental designs that track microbiota succession across developmental stages would provide a substantially more complete picture of the mineral–microbiota–host axis than current single-timepoint studies allow.

## Conclusion

This review has examined the bidirectional relationships between dietary minerals and gut microbiota in farmed aquatic species, integrating evidence from individual mineral studies (Fe, Zn, Mg, Se, Mn, Cu) and multi-mineral complex trials across a range of freshwater and marine organisms. Three overarching conclusions can be drawn from the collective evidence. First, mineral form is a primary ecological determinant of microbiota composition: organic chelates and nanoparticle forms consistently produce more targeted and beneficial microbiota shifts than inorganic salts, while partial substitution strategies combining both forms (approximately 50% organic replacement) appear to maximize outcomes in multi-mineral formulations (El-Sayed et al. 2023; Huang et al. 2025). Second, dose optimization is indispensable and species-specific; the non-linear dose–response relationships documented across all minerals reviewed here confirm that supplementation above or below a species-appropriate optimum risks compromising both host health and microbiota homeostasis (Lall and Kaushik 2021; Kong et al. 2022; Wei et al. 2025). Third, mineral–microbiota interactions are functionally inseparable from immune outcomes; the enrichment of lactic acid bacteria, enhanced goblet cell density, and increased mucosal immunoglobulin production observed following optimized mineral supplementation collectively constitute a nutritionally driven reinforcement of mucosal innate immunity (Salinas et al. 2011; Wei et al. 2021; He et al. 2025).

From a practical standpoint, while species- and stage-specific mineral requirement data remain incomplete, the reviewed studies collectively permit tentative guidance for formulators developing commercial diets for fish and shrimp. For individual trace minerals, optimal supplementation levels identified across species suggest approximate reference ranges: iron as Fe₂O₃ nanoparticles at 40–50 mg Fe/kg (Wei et al. 2025), zinc at 44–60 mg Zn/kg depending on form (Wei et al. 2021; Ma and Wang 2023), magnesium hydride at 20 mg/kg for warm-water carnivores (He et al. 2025), magnesium sulfate at 1.6–2.2 g/kg for freshwater shrimp (Kong et al. 2022), selenium nanoparticles at 1.5 mg/kg (Zahran et al. 2025), and organic manganese chelates at 0.01–0.022 g/kg (Xu et al. 2023). For combined mineral premixes, a partial substitution strategy replacing approximately 50% of inorganic minerals with organic chelates (e.g., MAAC or proteinate complexes) consistently yielded the most favorable outcomes across gut microbiota composition, growth performance, and feed efficiency (El-Sayed et al. 2023; Huang et al. 2025). These ranges align with nutritional requirements documented for bacterial growth promotion in culture media studies, where iron, zinc, and manganese in the low-mg/L range support beneficial species such as Lactobacillus and Bacillus, providing indirect validation of gut-targeted supplementation strategies. It is emphasized that these values should be regarded as starting points for species-specific optimization rather than universal prescriptions, and that inter-mineral antagonistic interactions—particularly Fe–Zn and Zn–Cu competition—must be accounted for in multi-mineral formulation design to avoid inadvertent microbiota dysbiosis.

### Future perspectives

Looking forward, several research priorities emerge as particularly urgent. The characterization of inter-mineral competitive dynamics (Fe–Zn, Zn–Cu, and their nanoparticle equivalents) at shared intestinal transporters must be addressed through controlled multi-element trials using isotope tracing, as these interactions have the potential to undermine single-mineral optimization efforts when translated to commercial multi-mineral premix formulations (Kwong 2024). Expanding species coverage to include commercially important coldwater salmonids, diadromous species, and emerging aquaculture candidates is equally pressing, given the documented influence of host feeding strategy, osmotic environment, and gut microbiota baseline composition on mineral–microbiota response profiles (Moroni et al. 2025; Llewellyn et al. 2014). Multigenerational studies assessing the epigenetic and reproductive consequences of nanoparticle mineral supplementation represent an additional priority, as the absence of such data constitutes a significant regulatory and commercial liability for the adoption of these novel mineral forms.

From a translational perspective, the integration of mineral nutrition with microbiome management through probiotic, prebiotic, or synbiotic co-supplementation represents the most promising near-term avenue for next-generation precision aquafeeds (Aydın and Çek-Yalnız 2019; Tay et al. 2025a, b). Mineral–prebiotic combinations that selectively amplify phytase-secreting commensals while maintaining luminal pH within an absorption-favorable range could allow dietary mineral inclusion levels to be reduced without compromising bioavailability, thereby simultaneously improving feed efficiency and reducing mineral discharge into receiving water bodies. The development of microbial biomarkers that reliably reflect the mineral nutritional status of individual fish or production cohorts would further enable adaptive feeding strategies responsive to real-time physiological demand rather than fixed dietary formulations. Such innovations, however, will only deliver their full potential if they are validated under commercial production conditions encompassing realistic stocking densities, thermal regimes, and water quality variability, since the controlled laboratory settings of all reviewed studies represent a significant departure from commercial aquaculture reality.

## Data Availability

No datasets were generated or analysed during the current study.
